# Cost-effectiveness of routine influenza vaccination program for individuals aged 65 years and older in Japan: Substituting standard-dose inactivated vaccine with high-dose inactivated vaccine

**DOI:** 10.1016/j.ijregi.2026.100858

**Published:** 2026-02-13

**Authors:** Shu-ling Hoshi, Xerxes Seposo, Masahide Kondo

**Affiliations:** 1 Department of Health Care Policy and Health Economics, Institute of Medicine, University of Tsukuba, Tsukuba, Ibaraki, Japan; 2 Department of Hygiene, Graduate School of Medicine, Hokkaido University, Sapporo, Japan

**Keywords:** Willingness-to-pay (WTP), Quality-adjusted life year (QALY), Incremental cost-effectiveness ratio (ICER), Decision-tree model, Monte Carlo simulations

## Abstract

**Objectives:**

We evaluated cost-effectiveness of influenza immunization programs for protection of Japanese elderly (aged ≥65 years) from influenza-related diseases by comparing high-dose inactivated influenza vaccine (HD-IIV3) to standard vaccines (SD-IIV3).

**Methods:**

Incremental cost-effectiveness ratio (ICER) from payers’ perspective was calculated. Variables used in the model were either calculated or extracted from literature. Quality-adjusted life years (QALYs) was used as measure of effectiveness. Cost per dose of HD vaccine was assumed to be four times of SD (JPY 1500/US$ 10).

**Results:**

In comparison to SD, HD gained more QALYs and reduced disease treatment costs, but these reduced costs did not offset vaccination cost. ICER for those aged ≥65 years was JPY 7,352,458/ US$ 49,016 per QALY, higher than the willingness-to-pay (WTP) threshold of JPY 5,000,000/US$ 33,333 per QALY. While ICER was JPY 50,477,31/US$ 33,652 for those aged ≥75 years. One-way sensitivity analysis revealed that relative vaccine effect against laboratory-confirmed influenza of HD to SD influenced the ICER the most. Monte Carlo simulations revealed that the probability of an ICER below WTP threshold was 20.9%.

**Conclusions:**

For a WTP of JPY 5,000,000/US$ 33,333 per QALY gained, if the price of HD was four times the current vaccine, the seasonal HD-IIV3 vaccination program was not cost-effective for those aged ≥65 years, while was cost-effective for those aged ≥75 years.

## Highlights

• Cost-effectiveness of high-dose influenza vaccine (HD) among persons aged ≥65 years was examined.
• Incremental cost-effectiveness ratios were presented not only for people aged ≥65 years but also for the following age groups: 65-69, 70-74, 75-79 and >80 years.
• HD vaccination program was cost-effective if the price of HD was less than 3.15 times that of standard vaccines.
• Age influences the incremental cost-effectiveness ratio, with the results being cost-effective only when the target age was ≥75 years.

## Introduction

Seasonal influenza is an acute respiratory infection caused by influenza viruses, with four types, namely, A, B, C, and D. Influenza A and B viruses circulate and cause seasonal epidemics [1]. Influenza spreads easily between people when they cough or sneeze and is common in all parts of the world. Most people recover from fever and other symptoms within a week without requiring medical attention, but it can cause severe illness or death, especially in people at high risk [1]. The elderly population, who show age-related declines in immune system function, have disproportionately high rates of seasonal influenza-related hospitalizations and deaths [[1], [2], [3]]. In industrialized countries, most deaths associated with influenza occur among people aged ≥65 years [1]. In the European Union, mortality rates of influenza-related diseases are 35 times higher in people aged ≥65 years than in those aged <65 years [4].

Vaccination is the best way to reduce disease burden. While vaccination varies from country to country, annual influenza vaccination is recommended for pregnant women, children, persons aged ≥65 years, individuals with chronic disease, and among healthcare workers [5]. Safe and effective vaccines have been used for more than 60 years with studies indicating that the vaccine is less effective in the elderly due to age-related immunosenescence [6]. Enhanced vaccines such as high-dose inactivated influenza vaccine (HD-IIV), adjuvanted IIV (adj-IIV), and recombinant IV (RIV) have been developed for people aged ≥60 years [7]. These vaccines provide higher immunogenicity and their relative vaccine efficacy/effectiveness (rVE) to standard vaccines (SD-IIV) have been well-documented [7].

Over 30 countries in the world have recommended three-valent or four-valent HD-IIV, i.e., HD-IIV3 or HD-IIV4 (administered intramuscularly, IM) to the elderly [8]. In the USA, influenza vaccine uptake rates among Medicare beneficiaries aged ≥65 years were estimated to be at 74%, 80%, and 81% between the 2017-2018 through 2019-2020 seasons. Among the three enhanced vaccines (HD-IIV3, aIIV3, and RIV4), HD-IIV3 usage accounted for about 70% [9]. In Canada, the National Advisory Committee on Immunization (NACI) recommends HD-IIV, adj-IIV, and RIV, over other influenza vaccines for adults aged ≥65 years [10]. In the UK, HD-IIV is one of the preferred influenza vaccines for adults ≥65 years [11]. Previous economic evaluation studies from high-income countries, such as France, Korea, Denmark, Norway, Sweden, the US, and Canada, reported cost-effective or cost-saving results of HD vaccination program compared to SD vaccination program; although most of these studies were industry-supported [[12], [13], [14], [15], [16], [17], [18], [19], [20], [21]]. In contrast, in Quebec, enhanced vaccines were not cost-effective (when rVE is 25% and the vaccine price differs from SD-IIV by CND$ 30) [10]. Economic analysis conducted by National Center for Immunization and Respiratory reported that 20% of scenarios were cost-saving, and about 50% of scenarios were between US$ 50,000 and US$ 195,000/QALY gained [9].

In Japan, the Preventive Vaccination Law was amended in 2001 to include influenza vaccine as a government-subsidized routine vaccination to be administrated subcutaneously (SC) for persons aged ≥65 years, and to those persons aged 60-64 years with heart, kidney, respiratory organ dysfunction, or immunodeficiency diseases. The locally produced SD-IIV3 (∼2014) and SD-IIV4 (2015∼) have been the only vaccines used in the immunization program. Regardless of the current immunization program in place, 63% of patients hospitalized for influenza in 2018-2019 were adults aged ≥60 years [22]. The social burden caused by influenza is considered to be substantial. On February 20, 2025, the Subcommittee on Vaccines agreed to prepare a fact sheet regarding HD-IIV for the elderly, with an eye toward its potential to substitute the current standard-dose routine vaccination program with the high-dose vaccine following the approval of HD-IIV4 on December 27, 2024. Similarly, a previous industry-financed study reported the cost-effectiveness of substituting SD vaccination program with HD vaccination program in Japan with an incremental cost-effectiveness ratio (ICER) at JPY 4,876,512/QALY at a willingness-to-pay (WTP) of JPY 5,000,000 per QALY [23]. Against this backdrop, and given that the Ministry of Health, Labour and Welfare (MHLW) has announced that trivalent influenza vaccines will be used from the 2025-2026 season onwards, we evaluated the value for money of influenza immunization programs for protection of Japanese elderly from influenza-related diseases by comparing HD-IIV3 (via IM) to SD-IIV3 (via SC) from an academic standpoint to inform decision-makers about the potential impact of switching SD-IIV to HD-IIV vaccination program.

## Methods

We conducted a cost-effectiveness analysis comparing the HD-IIV3 vaccination program to SD-IIV3 vaccination program. The target population was persons aged ≥65 years. A payers’ perspective lens (including the government, municipalities, vaccinees, patients and third-party payers) was considered in this study [24]. We used quality-adjusted life years (QALYs) as a measure of effectiveness, wherein the ICER was calculated as the cost difference divided by the effectiveness difference (QALY) between HD vaccination program and SD vaccination program. Since vaccination increases the chances of an individual to live up to one’s life expectancy, years of life gained resulting from vaccination was calculated. The efficiency of switching from SD influenza vaccination program to HD vaccination program is determined by whether the ICER is within the WTP threshold (JPY 5,000,000/QALY gained) or not. ICERs were also estimated for various age groups, namely, 65-69, 70-74, 75-79, and ≥80 years.

### Literature search

We searched the various databases for the parameters which were included in the modeling. Medline database, Igaku Chuo Zasshi database (a Japanese medical bibliographic database which contains over 10 million citations originating from Japan), MHLW Grant System, annual statistical reports published by the government, the Cochrane Database of Systematic Reviews, Health Technology Assessment database, the National Health Service, and Economic Evaluation Database were accessed. The subsequent model parameterization was specifically informed by variables from literature particularly those of local relevance, yielding robust yet locally relevant results.

### Models and variables

**Figure 1:**
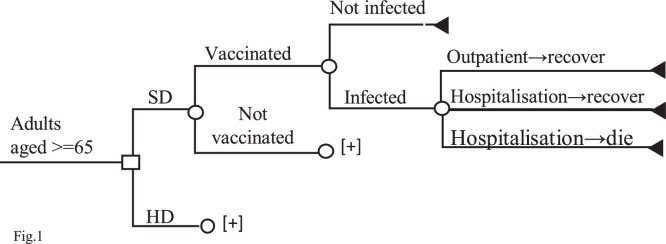
Decision tree model. □: decision node; 〇: chance node; ◁: terminal node

We developed a decision-tree model to describe the clinical courses that individuals follow in an influenza season, as shown in Figure 1. It has a decision node with two alternative programs and eight chance nodes in each program charted against the age groups of 65-69, 70-74, 75-79, 80-84, 85-89, 90-95, 95-99, and ≥100 years, with distributions based on the population estimate. These chance nodes were further followed by vaccination status and treatment courses of influenza (outpatient or hospitalization). We assumed that the eligible individuals who uptake SD would also uptake HD once the program was switched, thus we used the same uptake rate, at 57.8%, for both programs [25]. Adverse health effects due to vaccination were not included because there were no safety-related issues reported in administering HD-IIV4 via IM route compared to SD-IIV4 via SC route [26]. All the variables were shown on Table 1.

**Table 1:** Model parameters.

Variables		Value	Reference
Vaccine uptake		57.8%	[25]
Probabilities of an individual to receive outpatient or hospitalization (/100000)			[27]
Outpatient	aged 65-69, 70-74, 75-79, 80-84, 85-89, ≥90 years	3761, 3106, 2719, 2686, 2826, 3157	
Hospitalization		Mild: 66.6, 85.6, 122.2,186.9, 263.0, 324.7	
		Moderate: 36.5, 54.9, 90.5, 161.4, 272.6, 432.3	
		Severe: 7.9, 10.2, 15.5, 21.8, 29.8, 31.1	
Case-fatality rate among hospitalized patients (%)		(aged 65-69,70-74,75-79,80-84,85-89,90+)	[27]
Mild/moderate	aged 65-69, 70-74, 75-79, 80-84, 85-89, ≥90 years	2.6, 3.3, 4.3, 5.8, 8.2, 12.5	
Severe		25.4, 27.1, 30.8, 38.8, 43.2, 50.4	
Relative vaccine effectiveness of high dose vs standard dose		24.2%	[28,29]
Costs of vaccination (JPN)		Standard dose: 1500, High dose: 6000, Administration: 3930	[30]
Costs of disease treatment (JPN)			[3]
Outpatient	Aged 65-74/aged ≥75 years	12,702/ 15,259	
Hospitalization	Aged 65-74/aged ≥75 years	Mild: 616,152/461,737	
		Moderate: 670,350/ 557,330	
		Severe: 905,111/ 770,370	
Utility weights			[31]
Outpatient		0.738	
Hospitalization		Mild: 0.664, Moderate: 0.589, Severe: 0.336	
Mean influenza (days)		7	Assumed
Length of hospitalization (days)	Aged 65-74/75-84/≥85 years	Mild & moderate: 13/16/19	[32], assumed
		Severe: 19/21/25	[32], assumed

#### Probabilities

Probabilities of an individual to receive outpatient, hospitalization treatment (mild, moderate, and severe), and death from hospitalization were from Noda et al. [27]. The authors estimated these rates using the National Database of Health Insurance Claims and Specific Health Checkups of Japan (NDB; data from September 2017∼ August 2020). These were assumed to be the probabilities under the ongoing SD vaccination program, thus, the probabilities for an individual who received HD vaccine would be (1-rVE) multiplied by the probabilities described previously. In Noda et al. [27], influenza patients were identified by a definitive diagnosis of influenza (International Classification of Diseases, Tenth Revision [ICD-10] code: J09-J11) and/or a prescription of anti-influenza drugs (baloxaiv marboxil, laninamivir, oseltamivir, peramivir, zanamivir) between September 2017 and August 2020. In brief, J09 refers to influenza due to a specific identified influenza virus, J10 refers to influenza due to other identified influenza viruses, and J11 refers to influenza due to an unidentified influenza virus. The clinical courses in our study were defined following that of Noda et al. [27]. Below are the definitions of each state:

• Influenza patient: those diagnosed with influenza and/or prescribed anti-influenza medication.
• Hospitalization: all influenza patients who were hospitalized within 28 days of visit (patients originally hospitalized, nursing home residents, or outpatients were excluded).
• Moderate patient: hospitalized patient who received either nasal high flow, non-invasive positive pressure ventilation, or oxygen therapy within 28 days of the visit.
• Severe patient: hospitalized patient who needs intensive care unit or is on a ventilator.
• Influenza-related death: influenza patients died within 28 days after the index date and were identified by an algorithm to identify deaths based on information about diagnosis and procedural records.

#### Relative vaccine effectiveness of HD-IIV vs SD-IIV

Relative effectiveness (rVE) of HD-IIV vs SD-IIV against laboratory-confirmed influenza was set at 24.2%, based on 1) RCT studies [28,29], 2) a study that reported superior immunogenicity of HD-IIV4 administered via IM vs SD-IIV4 administered via SC [26], and 3) HD-IIV4 induced high immunogenicity compared with SD-IIV4, even though two of the four strains contained in HD-IIV4 (selected as per the recommendation of the World Health Organization and the United States Vaccines and Related Biological Products Advisory Committee) are different with strains in SD-IIV4 used in Japan (recommend by National Institute of Infectious Disease of Japan) [26].

#### Utility weights for estimating QALY

Utility weights used in this study were from Nakagawa et al. [31]. The authors reported eight health state utilities for influenza-related diseases among Japanese adults. We used the health state utilities assumed in their study since the health states that they identified were similar to ours. The utilities and their respective health state are as follows: 0.738 for outpatient, 0.589 for moderate hospitalized, 0.336 for severe hospitalized patients, and (0.738 + 0.589) x 0.5 for mild hospitalized patients. Hospital length were 19, 21, and 25 days for severe cases aged 65-74, 75-84, and ≥85 years, respectively, and 13, 16, and 19 days for mild and moderate cases, respectively [32].

#### Cost

Direct payments to health care providers by the government, municipalities, vaccinees, patients and third-party payers were estimated as costs, while non-direct medical costs related to the vaccination program were not included, because we assumed that the vaccination program will be within the public health services routine domain. Vaccine price per HD shot was assumed to be four times that of SD (JPY 1500/US$ 10; US$ 1 = JPY 150) based on the price of Fluzone ($14.34) and Fluzone high-dose ($59.4) as listed in the CDC price list (2025-2026 season) [30]. Fee for administering (including doctor’s fee for medical advice and technical fee) was JPY 3930/US$ 26.2. Treatment costs per outpatient visit (including test-related costs) were JPY 12,702/US$ 84.7 for those aged 65-74 years and JPY 15,259/US$ 101.7 for those aged ≥75 years. These figures were the weighted average of treatment costs for emergency and outpatient visits from Arashiro et al. [3]. In Japan, more than 99% of non-hospitalized influenza patients are reported to have outpatient visits, and the remainder are reported to have emergency room visits [3]. Treatment costs per hospitalization for those aged 65-74 and ≥75 years, respectively, were JPY 616,152/US$ 4107.7 and JPY 461,737/US$ 3078.2 per mild case, JPY 670,350/US$ 4469.0 and JPY 557,330/US$ 3715.5 per moderate case, and JPY 905,111/US$ 6034.1 and JPY 770,370/US$ 5135.8 per severe case. These data were derived from the study by Arashiro et al., which examined 30-day hospitalization costs for influenza patients, 30-day hospitalization costs for broadly defined influenza cases (including influenza or pneumonia), and 60-day hospitalization costs for broadly defined influenza [3]. The previously mentioned data were also consistent with that of Hagiwara et al., which analyzed 372,356 clinically diagnosed influenza cases and 31,122 laboratory-confirmed influenza cases from nine seasons (2010/21-2018/19) [32].

#### Discounting

Health benefits accrued after the first year (life expectancy for survival individuals) were discounted at an annual rate of 3%, with no discounting on costs since all costs were assumed to be accrued within the first year.

### Sensitivity analyses

We performed one-way sensitivity analyses in all variables to appraise the ICER’s stability with the assumptions made in our economic model and to explore the impact of each variable relative to each other. Probabilistic sensitivity analyses (PSA) with 10,000 Monte Carlo simulations, were also performed. Lower and upper limits were adopted from the 95 confidence interval of the variables. If lower and upper limits were not available, we assumed a ±50% of base-case value for cost items, ±30% for probabilities, and ±20% for utility weights, with a ceiling for the upper limit set not higher than 1. We used a triangular distribution for each variable.

## Results

### Base-case analysis

Table 2 shows the costs, effectiveness, and ICER at base-case analysis. In comparison to SD, the estimated incremental effects of HD for individuals aged ≥65 years were 0.00032 QALYs per person. HD reduced disease treatment costs, but these reduced costs did not offset vaccination costs, which means that HD vaccination program gained more QALYs with more costs. Majority of treatment savings from HD vaccination program came from reduced hospital admissions, which accounted for 78.7% of the total reduction. ICER for those aged ≥65 years was JPY 7,352,458/US$ 49,016 per QALY. Table 3 shows the ICERs by age group at JPY 6,349,908/US$ 42,333 and JPY 5,047,731/US$ 33,652 per QALY for those aged ≥70 years, ≥75 years, respectively. Subsequently, when compartmentalized into 65-69, 70-74, 75-79, and ≥80 years age groups, ICERs were JPY 17,772,436/US$ 118,483, JPY 14,008,592/US$ 93,391, JPY 9,315,246/US$ 62,102 and JPY 3,886,141/US$ 25,908, per QALY, respectively.

**Table 2:** Results of base-case analysis for persons aged ≥65 years.

	Vaccination cost	Treatment cost		Total cost	Effectiveness	ICER
		Outpatient	Hospitalization			
	(JPY)	(JPY)	(JPY)	(JPY)	(QALY)	(JPY/QALY)
Standard dose ≥65 years	3139	429	1585	5152	12.07920	-
High dose ≥65 years	5740	369	1365	7474	12.07952	7,352,458

**Table 3:** Costs, effectiveness, and ICERs by age groups.

	Total cost	Effectiveness	ICER
	(JPY)	(QALY)	(JPY/QALY)
SD 65-69	4343	16.71333	-
HD 65-69	6777	16.71347	17,772,436
SD 70-74	4520	14.39013	-
HD 70-74	6930	14.39031	14,008,592
SD 75-79	4742	11.97446	-
HD 75-79	7120	11.97471	9,315,246
SD ≥80	6308	7.82944	-
HD ≥80	8469	7.82999	3,886,141
SD ≥65	5152	12.07920	-
HD ≥65	7474	12.07952	7,352,458
SD ≥70	5357	10.90341	-
HD ≥70	7651	10.90377	6,349,908
SD ≥75	5725	9.37219	-
HD ≥75	7967	9.37264	5,047,731

HD, high dose; ICER, incremental cost-effectiveness ratio; SD, standard dose.

### Results of sensitivity analyses

#### One-way sensitivity analyses

**Figure 2:**
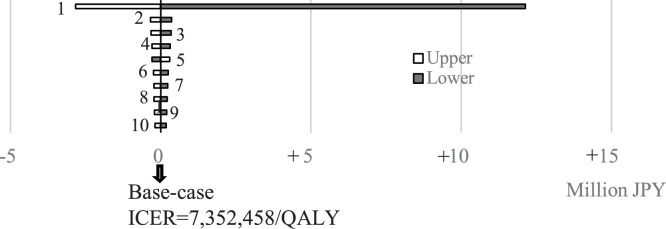
Results of one-way sensitivity analysis (only the top 10 were shown). 1. Relative vaccine effectiveness of high dose-influenza vaccine vs standard dose-influenza vaccine against laboratory-confirmed influenza 2. Probability of death from influenza (aged ≥90 years) 3. Probability of death from influenza (aged 85-89 years) 4. Probability of death from influenza (aged 80-84 years) 5. Utility for outpatient treatment 6. Illness day (outpatient) 7. Probability of hospitalization (moderate case; aged ≥90 years) 8. Probability of death from influenza (of not severe case; aged 75-79 years) 9. Probability of hospitalization (moderate case, aged 85-89 years) 10. Probability of hospitalization (mild case, aged 85-89 years) ICER, incremental cost-effectiveness ratio; QALY, quality adjusted life years.

The largest and smallest ICERs, JPY 19,495,659/US$ 129,971 and JPY 4,531,569/US$ 30,210 per QALY, were observed at the lower and the upper limits of rVE against laboratory-confirmed influenza (9.7% and 36.5%), respectively. The lower limit yielded an increase of JPY 12,143,201/US$ 80,955 per QALY, whereas the upper limit yielded a decrease of JPY 2,820,889/US$ 18,806 per QALY compared to the base-case ICER. Other variables had minimal impact on the ICER (Figure 2). The rVE against hospitalization was not included in base-case analysis, however, if incorporated (rVE = 8%), the ICER would decrease to JPY 5,804,942/US$ 38,700 per QALY. One-way sensitivity analysis on HD vaccine price indicated that if the price of HD was less than 3.15 times that of SD, the ICER per QALY would be less than JPY 5 million/QALY.

#### Probabilistic sensitivity analysis

**Figure 3:**
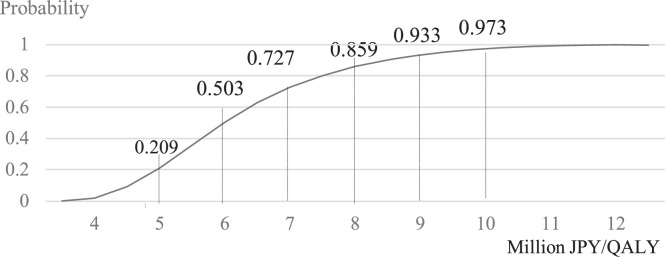
Results of probabilistic sensitivity analyses: acceptability curve of 10,000 incremental cost-effectiveness ratios generated by Monte Carlo simulations. QALY, quality adjusted life years.

Figure 3 shows the acceptability curve of 10,000 ICERs generated by Monte Carlo simulations. The probability that ICER was under JPY 5,000,000/US$ 33,333, JPY 6,000,000/US$ 40,000, JPY 7,000,000/US$ 46,667, JPY 8,000,000/US$ 53,333, JPY 9,000,000/US$ 60,000 and JPY 10,000,000/US$ 66,667 per QALY gained, was at 20.9%, 50.33%, 72.7%, 85.9%, 93.3%, and 97.3%, respectively.

## Discussion

Motivated with the recent approval of HD-IIV for the prevention of influenza for persons aged ≥65 years in Japan, we examined the cost-effectiveness potential of substituting SD-IIV3 vaccination program with HD-IIV3 vaccination program. Results showed that while HD vaccination program reduced disease treatment costs and gained more QALYs, it was not able to offset the vaccination costs. Base-case ICER was at JPY 7,352,458/US$ 49,016 per QALY gained, which was higher than the WTP threshold of Japan (JPY 5,000,000/US$ 33,333 per QALY gained). The ICERs by age group ranged from JPY 3,886,141/US$ 25,908 per QALY (≥80 years) and JPY 17,772,436/US$ 118,483 per QALY (65-69 years old). It is noteworthy that the older target age group tended to have smaller ICER. One-way sensitivity analyses showed that rVE against influenza was the key variable that substantially affected ICER. Among the results of one-way sensitivity analyses, the lower and upper limits of rVE yielded the highest and lowest ICER at JPY 19,495,659/US$ 129,971 and JPY 4,531,569/US$ 30,210 per QALY, respectively. One-way sensitivity analysis on vaccine price showed that if the price of HD was less than 3.15 times that of SD, the ICER would be less than the WTP. PSA results showed that the probability that ICER be under JPY 5,000,000/US$ 33,333, JPY 6,000,000/US$ 40,000, JPY 7,000,000/US$ 46,667, JPY 8,000,000/US$ 53,333, JPY 9,000,000/US$ 60,000 and JPY 10,000,000/US$ 66,667 per QALY gained, was at 20.9%, 50.33%, 72.7%, 85.9%, 93.3%, and 97.3%, respectively.

Several studies have evaluated the cost-effectiveness of switching from SD influenza vaccination program to advanced influenza vaccines (including high-dose vaccines, influenza vaccines using adjuvants, reconstituted influenza vaccines, etc.). Particularly, the studies that we have reviewed, which were mostly from high-income countries, exhibited a limited comparison between HD vaccination program and SD vaccination program with a payers’ perspective (or perspective of health care system), similar to the current study. A total of 12 cost-effectiveness analyses (CEAs) from 10 studies were identified, with six studies (seven CEAs) reporting ICERs of HD-IIV4 vs SD-IIV4 (one each from The Netherlands, Belgium, Finland, Korea and two from France), four reporting ICERs of HD-IIV3 vs SD-IIV3 (one from Canada and three from USA), and one not specifying the valent of the vaccine (Japan) [[12], [13], [14], [15], [16], [17], [18], [19], [20],^23^]. Five studies (seven CEAs) had their first authors [14,[18], [19], [20],^23^], and three studies had their co-authors who were from industry [13,15,16]. As to the other two studies, one was industry funded and supported [12], one reported a conflict of interest due to the role of the industry in the conduct of the study [17]. These studies differed substantially in several respects, such as the (i) price ratio of HD to SD (ranging from 1.4 to 6.0 times), (ii) modeling approaches (one dynamic model, two trial-based “piggyback” CEA, and the remainder decision-tree models), (iii) definitions of influenza-related disease burden, and (iv) discount rate applied to health benefits beyond the second year (ranging from 1.5% to 5%). Despite these differences, all 10 studies (12 CEAs) found HD to be an economically attractive alternative to SD. Two trial-based CEAs reported cost-savings results [16,18], while the others reported these to be cost-effective. The ICER/WTP ratios of the nine studies ranged from 0.03 (Belgium) to 0.98 (Japan) (see Supplementary). In contrast, our estimated ICER was higher than the assumed WTP, which differs from the findings of the previous studies. This discrepancy is likely attributable to the difference in the definitions and scope of hospitalization and mortality outcomes. Both our study and that of the previous Japanese study restricted the analysis to hospitalizations and deaths directly attributable to influenza as defined by ICD-10 codes J09-J11. In contrast, most overseas studies incorporated hospitalizations for potentially influenza-associated cardiopulmonary conditions, which can substantially reduce the ICER estimates [[14], [15], [16],[18], [19], [20]].

Notably, CEA results reported by overseas official bodies differ from those of the aforementioned previous studies. For example, the NACI’s Advisory Committee Statement concluded that in Quebec the use of enhanced vaccines (rVE = 25%, vaccine price = CND$ 30 + SD vaccine) did not appear to be cost-effective [10]. Similarly, the National Center for Immunization and Respiratory reported that among the various modeling assumptions, 20% of scenarios were cost-saving, and about 50% of the scenarios were between US$ 50,000 and US$ 195,000 per QALY gained [9]. Studies reported some degree of industry involvement, such as industry-affiliated authorship, funding or support were found to have a lower ICER/WTP ratio compared to studies without industry involvement. This tendency has also been observed in a systematic review evaluating the effectiveness of other vaccination programs [33].

It is difficult to compare CEA results from different healthcare systems or even CEA results from the same healthcare system with different model designs and variables. We briefly charted our study with the previous CEA study in Japan (by Courville et al.) [23]. The definition of influenza disease and the rVE for influenza prevention were the same between our study and that of the previous studies. The vaccine price ratio for HD/SD in our study was lower than that in the previous study (four times SD vs 4.4 times SD) and the disease treatment costs were lower in our study. Estimation of QALY loss was also different. In our study, QALY losses were estimated by using utility weights and duration of health states (both were based on domestic studies), while the previous study cited QALY loss value directly from CEA of overseas studies. Our study assumed that the probabilities of outpatient visits and hospitalization reported by Noda et al. [27] represented the disease burden under the ongoing SD vaccination program. We then applied the rVE to estimate the burden under the HD vaccination program. In contrast, the previous study estimated the burden under the SD vaccination program using attack rate, outpatient visit probability, and VE of SD vaccine.

Our study has limitations. Firstly, the influenza disease burden in our study was from Noda et al. [27], thus the limitations listed in the previous study are also the limitations in the current study. Such as (i) while efforts have been made to eliminate “convenient diagnosis” or “disease names for insurance claim” (these are not true disease names) from NDB, complete elimination is difficult, and, (ii) hospitalization and death were defined as cases within 28 days of the patient being diagnosed with seasonal influenza, however, hospitalization and death for reasons other than influenza could also be included. Second, in Japan, influenza vaccinations are currently administered subcutaneously, but the rVE reported in this study was calculated by comparing HD (IM) and SD (IM), while one study reported that subcutaneous administration has low immunogenicity [34]. Therefore, when comparing HD vaccines (IM) and SD vaccines via SC, the rVE may be higher than 24.2%, which could result in a decrease in ICER. Another issue related to rVE is that, although the study demonstrates that high-dose influenza vaccines consistently confer greater preventive efficacy than SD-IIV [35], irrespective of the circulating influenza strain or antigenic match status, regional mismatches between vaccine strains and circulating viruses may still influence observed effectiveness. Third, influenza cases without a medical consultation (i.e., self-medication) were not considered, as data quantifying this impact could not be identified. Fourth, our model does not categorize the target population into high-risk and non–high-risk groups based on individual health status, as the initial studies reporting incidence rates did not present data separately for these two categories. Also, we adopt same rVE to all age, which is in contrast to a previous study that evaluated the effect of age on rVE of HD vs SD influenza vaccine (from 2012 to 2018 season) reported that there had been a slightly increasing trend in rVE with age in all seasons, especially was consistently more effective than SD for adults aged ≥85 years [34]. Using a variable rVE with age would tend to lower the ICERs. Fifth, we did not account for any indirect effects because there is limited evidence about how SD or HD vaccines will interrupt transmission. Sixth, our model did not account for long-term disability that could result from influenza complications. The presence of associated comorbidities in older adults has been shown to increase the odds of intensive care unit admission and severe and fatal infections following influenza infection by up to 7-fold, with particular risk associated with immunodeficiency, respiratory disease, and chronic liver disease identified in different population groups in Japan [32]. Sixth, the incidence rate reported by Noda et al. is based on the period when the quadrivalent vaccine was used. Finally, we acknowledge that dynamic models can capture herd effects and time-varying transmission dynamics. However, given data limitations in Japan and the seasonal nature of influenza, we considered that a decision-tree model is deemed to be appropriate for this analysis. Most prior influenza CEAs have used decision-tree models, and only one study in our review employed a dynamic approach.

Despite these limitations, our study has strengths. First, except for rVE and the price of HD, all inputs to the model are based on domestic studies. In particular, probabilities of an individual to receive outpatient/hospitalization treatment and probabilities of mild/moderate/severe patients and case-fatality rates among hospitalized patients were from a study using real-world data obtained over multiple influenza seasons (NDB; data from September 2017 to August 2020). The use of real-world evidence is preferentially recommended by some National Immunization Technical Advisory Groups [36].

## Conclusion

For a WTP of JPY 5,000,000/US$ 33,333 per QALY gained, if the price of HD was four times the current vaccine, the seasonal HD-IIV3 vaccination program was not cost-effective. The rVE and vaccine price may drive a difference to the ICER. In light of uncertainty surrounding vaccine performance across influenza seasons, continuous monitoring and real-world data collection are important, particularly in the context of antigen drift and potential vaccine-virus mismatch. Given that the employment rate of older people aged ≥65 years is on the increase (25.2% in 2023), especially as those aged 65-69 years account for 52.0% of the 25.2%, if the productivity losses due to influenza were to be considered, ICER is expected to be more favorable.

## Declaration of competing interest

The authors have no competing interests to declare.

## Appendix Supplementary materials

Image, application 1
