# Supplementary material for: Cost-effectiveness of routine influenza vaccination program for individuals aged 65 years and older in Japan: Substituting standard-dose inactivated vaccine with high-dose inactivated vaccine
Source: IJID Reg. 2026 Feb 13;18:100858. doi: 10.1016/j.ijregi.2026.100858 (PMC12992081; doi:10.1016/j.ijregi.2026.100858)
Supplement: Supplementary file 1 [file mmc1.pptx]

## Slide 1
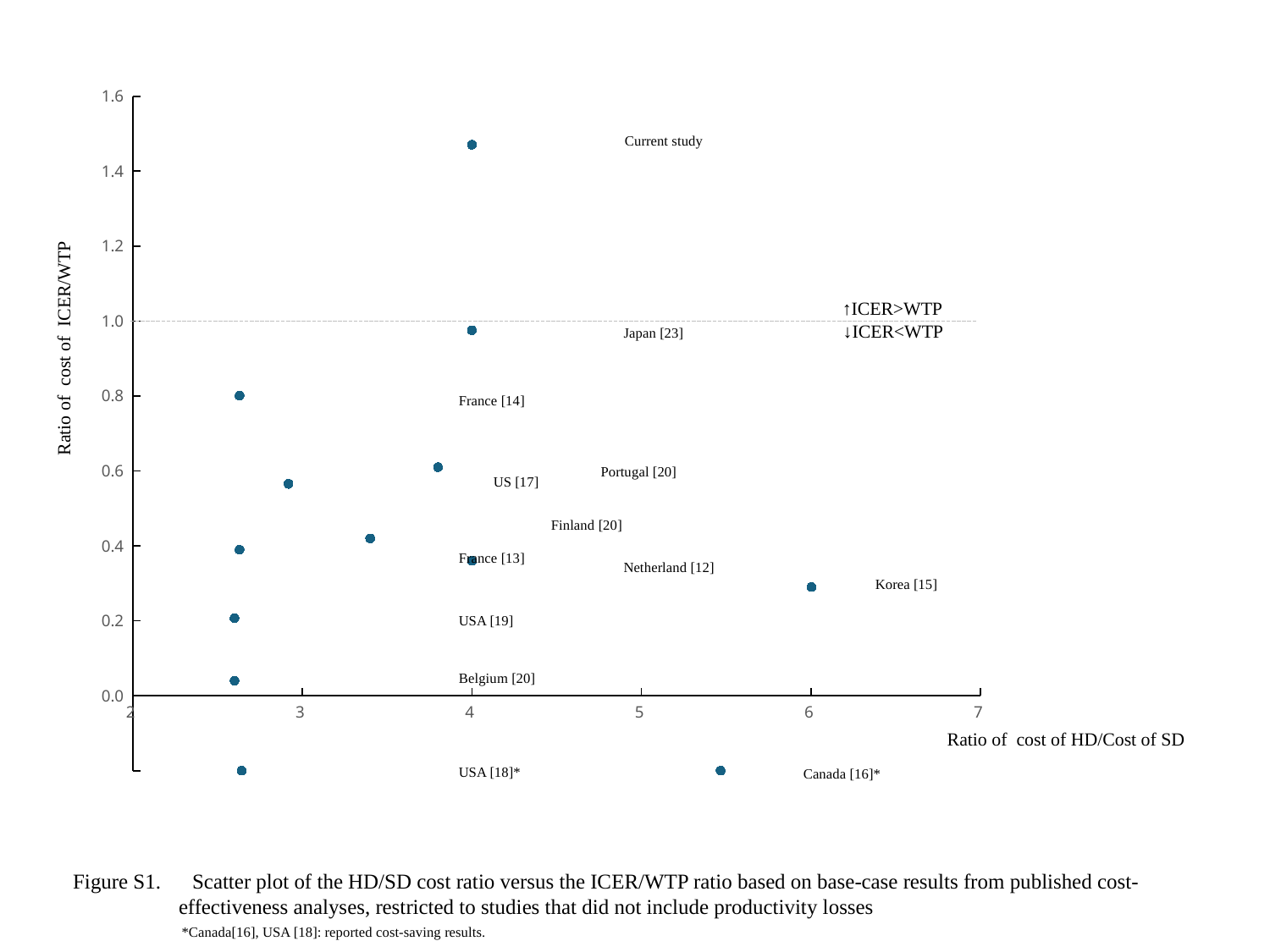

### Chart
| Category | |
|---|---|Current study
↑ICER>WTP
Ratio of cost of ICER/WTP
↓ICER<WTP
Japan [23]
France [14]
Portugal [20]
US [17]
Finland [20]
France [13]
Netherland [12]
Korea [15]
USA [19]
Belgium [20]
Ratio of cost of HD/Cost of SD
USA [18]*
Canada [16]*
Figure S1.　Scatter plot of the HD/SD cost ratio versus the ICER/WTP ratio based on base-case results from published cost-
 effectiveness analyses, restricted to studies that did not include productivity losses
*Canada[16], USA [18]: reported cost-saving results.
